# Ear-EEG Measures of Auditory Attention to Continuous Speech

**DOI:** 10.3389/fnins.2022.869426

**Published:** 2022-05-03

**Authors:** Björn Holtze, Marc Rosenkranz, Manuela Jaeger, Stefan Debener, Bojana Mirkovic

**Affiliations:** ^1^Neuropsychology Lab, Department of Psychology, University of Oldenburg, Oldenburg, Germany; ^2^Neurophysiology of Everyday Life Group, Department of Psychology, University of Oldenburg, Oldenburg, Germany; ^3^Division Hearing, Speech and Audio Technology, Fraunhofer Institute for Digital Media Technology IDMT, Oldenburg, Germany; ^4^Research Center for Neurosensory Science, University of Oldenburg, Oldenburg, Germany; ^5^Cluster of Excellence Hearing4all, University of Oldenburg, Oldenburg, Germany

**Keywords:** around-the-ear EEG, cEEGrid, auditory attention, speech envelope tracking, intersubject correlation (ISC), spectral entropy, auditory attention decoding (AAD)

## Abstract

Auditory attention is an important cognitive function used to separate relevant from irrelevant auditory information. However, most findings on attentional selection have been obtained in highly controlled laboratory settings using bulky recording setups and unnaturalistic stimuli. Recent advances in electroencephalography (EEG) facilitate the measurement of brain activity outside the laboratory, and around-the-ear sensors such as the cEEGrid promise unobtrusive acquisition. In parallel, methods such as speech envelope tracking, intersubject correlations and spectral entropy measures emerged which allow us to study attentional effects in the neural processing of natural, continuous auditory scenes. In the current study, we investigated whether these three attentional measures can be reliably obtained when using around-the-ear EEG. To this end, we analyzed the cEEGrid data of 36 participants who attended to one of two simultaneously presented speech streams. Speech envelope tracking results confirmed a reliable identification of the attended speaker from cEEGrid data. The accuracies in identifying the attended speaker increased when fitting the classification model to the individual. Artifact correction of the cEEGrid data with artifact subspace reconstruction did not increase the classification accuracy. Intersubject correlations were higher for those participants attending to the same speech stream than for those attending to different speech streams, replicating previously obtained results with high-density cap-EEG. We also found that spectral entropy decreased over time, possibly reflecting the decrease in the listener’s level of attention. Overall, these results support the idea of using ear-EEG measurements to unobtrusively monitor auditory attention to continuous speech. This knowledge may help to develop assistive devices that support listeners separating relevant from irrelevant information in complex auditory environments.

## Introduction

In everyday complex auditory scenes, one fundamental question to be answered is how the brain manages to select relevant and neglect irrelevant information. Although many studies on auditory attention have contributed to this question, most of them have been conducted in highly controlled laboratory settings using discrete and artificial stimuli. Two recent advances have opened up the possibility of measuring brain responses to natural stimuli in everyday life. First, the development of small and portable measurement devices has made it possible to measure brain activity outside of the lab (e.g., Debener et al., 2012). Second, methods have been developed to analyze the neural processing of natural and continuous stimuli such as speech (Hamilton and Huth, 2020). Here, we investigate the potential of combining these two developments to eventually measure attentional processes unobtrusively.

**FIGURE 1:**
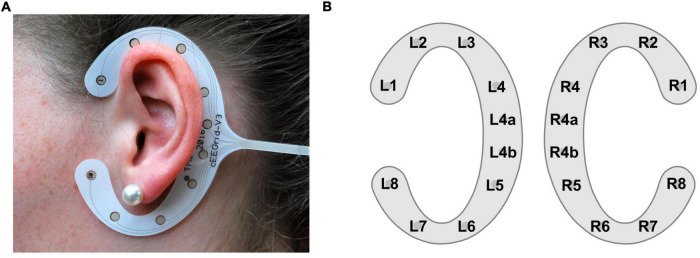
cEEGrid illustration. **(A)** A cEEGrid attached with double-sided adhesive around the left ear. **(B)** cEEGrid channel layout consisting of a pair of cEEGrids, one for the left and one for the right ear. Each cEEGrid comprises 10 electrodes. Electrodes R4a and R4b serve as ground and analog reference, respectively. In the analysis, data were re-referenced to the half of channel L4b (algebraic linked mastoids). To keep the number of channels symmetrical between the left and right cEEGrid channel L4a was removed in the analysis.

Electroencephalography (EEG) is a popular method to non-invasively measure human brain electrical activity by placing electrodes on the scalp. Traditional EEG as used in most laboratories require caps or nets to position electrodes on the scalp, which is not feasible for EEG acquisition in everyday life (Bleichner and Debener, 2017). For unobtrusive EEG acquisition, small and near-invisible approaches are preferred to not disturb natural social interaction. This demand has led to the development of in-ear EEG (Looney et al., 2012), and around-the-ear EEG solutions (Debener et al., 2015), where electrodes are placed inside the outer ear canal or around the ear, respectively. The cEEGrid is one around-the-ear EEG solution – a c-shaped flex-printed sensor array comprising 10 electrodes (Figure 1A). In the current study, we used the cEEGrid as it provides larger inter-electrode distances compared to in-ear EEG, leading to an increase in the measured EEG amplitudes (Bleichner and Debener, 2017) and better sensitivity to distant contributions (Meiser et al., 2020).

Debener et al. (2015) and Bleichner et al. (2016) already provided evidence that attentional processes can be captured with cEEGrids but used event-based analyses and time-domain trial averaging instead of measuring the neural response to continuous stimuli. Here we investigated three methods which cannot only analyze the neural processing of continuous speech but have also been shown to be sensitive to attentional effects. We evaluated their feasibility to capture neural effects of auditory attention when using around-the-ear EEG.

The first method is speech envelope tracking, which refers to the neural tracking of the slow amplitude fluctuations, i.e., the envelope, of speech (Aiken and Picton, 2008). When presented with more than one speaker at the same time, the listener’s neural signal correlates more strongly with the speech envelope of the attended than of the ignored speaker(s) (Power et al., 2012; Zion Golumbic et al., 2013). Based on this observation, many studies have been conducted to decode the attended among all present speakers from the listeners’ EEG (Mirkovic et al., 2015; O’Sullivan et al., 2015). Therefore, this method is often referred to as auditory attention decoding (AAD). However, we will not use this term, because in principle all three methods introduced here aim at decoding the listener’s attention. One of the main potentials of speech envelope tracking is that it can help to develop neuro-steered hearing aids that first identify and then enhance the attended speaker (Geirnaert et al., 2021b). This could have a tremendous impact for hearing-impaired listeners who have difficulties listening to one speaker in the presence of background noise (Shinn-Cunningham and Best, 2008).

The second method is known as intersubject correlations (ISCs). This method is based on the observations that individuals who are exposed to the same stimulus show similar spatiotemporal brain activity (Hasson et al., 2004; Dmochowski et al., 2012, for more recent reviews see Zhang, 2018 and Nastase et al., 2019). More recently, this approach has been adopted to attention research. When selectively attending to one of two simultaneously presented audio streams, ISCs of EEG signals were found to be higher for those participants attending to the same stream than for those attending to different streams (Stuldreher et al., 2020; Rosenkranz et al., 2021). Moreover, Rosenkranz et al. (2021) showed that the magnitude of participants’ ISCs with others attending to the same audio stream were positively correlated with the participants’ attentional effect observed in speech envelope tracking. Thus, the strength by which an individual’s EEG signal correlates with others attending to the same stimulus reflects the individual’s ability to selectively attend to the target stimulus and ignore the distracting stimulus. Regarding future application, this method could be of value in classroom scenarios (Poulsen et al., 2017; Janssen et al., 2021). For instance, this method could support students who have difficulties in focusing on the lecture content or support lecturers by identifying settings in which lectures are most effective (Brouwer et al., 2019).

The third method is spectral entropy. Spectral entropy characterizes the structure of an EEG spectrum (Viertiö-Oja et al., 2004) and has been proposed as a measure of attention. A high spectral entropy indicates an equally distributed EEG spectrum. This means that the power in each frequency band is very similar, whereas a low spectral entropy indicates an EEG spectrum in which the power is concentrated in one frequency band (Lesenfants and Francart, 2020). Lesenfants et al. (2018) found increased spectral entropy when participants actively attended to a stimulus compared to when they did not attend to the presented stimulus. In a consecutive study, Lesenfants and Francart (2020) showed that speech envelope tracking of a single speaker was increased during periods of high spectral entropy. Based on these findings, the authors concluded that high spectral entropy indicates high levels of attention. It is important to note that compared to speech envelope tracking and ISC, spectral entropy is not used to draw conclusion about one’s direction of attention. Instead, spectral entropy may be an informative measure in scenarios where it is important to monitor one’s level of attention, for example, when driving a car.

The aim of the current study was to test whether speech envelope tracking, ISCs, and spectral entropy capture effects of auditory attention to ongoing natural stimuli when unobtrusive around-the-ear EEG acquisition is used. To this end, we analyzed listeners’ brain activity captured with cEEGrids while they attended to one of two simultaneously presented, continuous speech streams. As speech envelope tracking has previously been performed on cEEGrid data but yielded rather low accuracies in identifying the attended speaker (Mirkovic et al., 2016; Nogueira et al., 2019), we explored the effect of artifact correction and of individualizing decoding models on the accuracy to identify the attended speaker.

## Materials and Methods

### Participants

In the current study, two previously recorded, unpublished cEEGrid datasets were combined. The cEEGrid datasets were each concurrently recorded with cap EEG. The corresponding cap EEG datasets were originally used in Jaeger et al. (2020) and Holtze et al. (2021), respectively, and later jointly used in Rosenkranz et al. (2021). Here, we only considered the cEEGrid datasets. From Jaeger et al. (2020), five out of the 20 participants had to be excluded due to data loss during the cEEGrid recording. From Holtze et al. (2021) all 21 participants could be included, resulting in a total of 36 participants (mean age 23.6 years, 25 females) in the current study. All participants were native German speakers, had normal hearing based on audiometric thresholds of 20 dB HL or better in both ears at octave frequencies from 250 Hz to 8 kHz (Holmes and Griffiths, 2019), and reported no psychological or neurological condition. Both original studies were approved by the local ethics committee (University of Oldenburg, Germany, Jaeger et al., 2020, Drs.Nr.27/2018; Holtze et al., 2021, Drs.EK/2019/006). All participants signed written informed consent before taking part in the respective study and received monetary reimbursement afterward.

### Task and Stimuli

Participants were comfortably seated in a dimly lit and sound-attenuated booth. They were instructed to attend to one of two simultaneously presented audio books and had to keep their attention on the same audio book throughout the experiment. To motivate participants and to make sure that they attended to the instructed audio book, participants had to answer questions related to the content of the to-be-attended audio book. Each audio book was narrated by a different male speaker (for further details see Mirkovic et al. (2016) where these stimuli were originally used). In Jaeger et al. (2020) each audio book was presented *via* a free-field loudspeaker located to the front-left (–45°) and front-right (+45°) side of the participant, respectively. In Holtze et al. (2021) the audio books were presented *via* earphones while the audio books were spatially separated at an angle of ±30° in azimuth using a head related transfer function (Kayser et al., 2009). Thus, in both studies, one audio book appeared to originate from the front left of the participant and the other one from the front right. The same audio books were used in both studies. The mode of presentation did not seem to affect the neural processing of the audio books as shown in Rosenkranz et al. (2021), where the cap-EEG data from Jaeger et al. (2020) and Holtze et al. (2021) were jointly analyzed. In both studies the audio books were presented in blocks of 10 min. In the Jaeger study the experiment consisted of six 10-min blocks while in the Holtze study it consisted of five 10-min blocks. Within the first 10-min block, both audio books were presented at equal volume in both studies. In the Jaeger study this was maintained for all remaining blocks. In the Holtze study only in two out of the remaining four blocks both audio books were presented at equal volume while in the other two blocks the to-be-attended audio book was enhanced. To keep the equal volume aspect constant across both studies, in the current study we only included the three blocks from the Holtze study where both audio books were presented equally loud. To keep the amount of data per participant constant across studies, we also selected only three blocks from the Jaeger study. This always included the first 10-min block plus two from the remaining blocks. Due to technical issues the cEEGrid data of some participants in the Jaeger study were not recorded during all blocks. For those participants where only three blocks were available, we used those. When more than three blocks were available, we pseudo-randomly selected two blocks such that blocks 2–5 were evenly represented across participants. Thus, in the current study we used 30 min of data per participant, which always included the first 10-min block.

### Data Acquisition

The cEEGrid recording procedure was identical for both original studies. For better electrode to skin conductance, the skin around the ears was prepared with abrasive gel (Abralyt HiCl, Easycap GmbH, Hersching, Germany) and cleaned with 70% alcohol. Thereafter, a small amount of abrasive gel was placed on the cEEGrid electrodes (TMSI, Oldenzaal, Netherlands; Debener et al., 2015) before it was attached with double-sided adhesive around the ear. Impedances were kept below 20 kΩ. Each participant was equipped with two cEEGrids, one around the left and one around the right ear. Electrodes R4a and R4b of the right cEEGrid served as ground and reference, respectively (Figure 1B). The two cEEGrids were connected to a 24-channel mobile amplifier (SMARTING, mBrainTrain, Belgrade, Serbia) which transmitted the data *via* Bluetooth to a recording computer. The cEEGrid data were acquired with a sampling rate of 500 Hz. The transmitted cEEGrid data as well as the onset markers of the 10-min blocks were integrated using the Lab Recorder software based on the Lab Streaming Layer^1^ to time synchronize these data streams (Mullen et al., 2015).

### Data Analysis

#### Preprocessing

All analysis steps were performed in MATLAB (R2019b, The Math-Works Inc., Natick, MA, United States), using custom scripts^2^. The cEEGrid data were processed with EEGLAB (version 2020.0; Delorme and Makeig, 2004) using the cEEGrid EEGLAB plugin^3^ (version 0.9). To account for the constant delay between the onset markers of the 10-min blocks and the corresponding EEG, we presented 20 beep tones to the participant prior to the experiment. We then computed the grand average event-related potential (ERP) in response to these beep tones and shifted the cEEGrid data to align the N1 latency of the cEEGrid data to the N1 latency observed in the cap-EEG, which in turn had been corrected based on a timing test. This resulted in a time delay of 54 ms for the Jaeger study and 70 ms for the Holtze study. The difference can be explained by the different audio presentation setups as described above (for details see Jaeger et al., 2020 and Holtze et al., 2021). Then, the cEEGrid data were re-referenced to algebraically linked mastoids by re-referencing the data to the half of channel L4b. To keep the cEEGrid layout symmetrical between the left and right side, channel L4a was removed, leaving 16 cEEGrid channels per participant (Figure 1B) (Debener et al., 2015).

#### Artifact Correction

Artifact correction was performed using artifact subspace reconstruction (ASR; Mullen et al., 2015), implemented in the EEGLAB plugin clean_rawdata (version 2.4). ASR identifies and reconstructs segments containing artifacts based on the statistics of artifact-free calibration data. In the current study, no explicit calibration data was provided, instead the plugin function automatically selected artifact-free calibration data from the entire recording. The clean_rawdata wrapper function consists of multiple sub-functions. The sub-functions clean_flatlines and clean_channels were not used, to keep the number of channels constant for all participants, and because the interpolation of removed cEEGrid channels may not produce reliable results (cf. Kang et al., 2015). As ASR requires high-pass filtered data (Mullen et al., 2015), we used the clean_drift function within clean_rawdata with the default high-pass transition band from 0.25 to 0.75 Hz. As cutoff parameter for the clean_asr function we used a rather liberal value of 10, as cutoff values below 10 may be prone to remove brain data (Chang et al., 2018). The function clean_windows, which removes data segments that still contain artifacts after performing ASR, was not used as continuous signals were required for the analyzes.

#### Speech Envelope Tracking

As mentioned, speech envelope tracking has previously been implemented with cEEGrid data but yielded rather low accuracies when the aim was to identify the attended speaker (Mirkovic et al., 2016: 69.33% with 50 one-min-segments per participant; Nogueira et al., 2019: 59.79% with 48 one-min segments per participant). Therefore, we systematically investigated two adaptations of the analysis pipeline used in Mirkovic et al. (2016), with the goal of increasing the classification accuracy. For a better understanding we now first describe how we implemented the analysis pipeline described in Mirkovic et al. (2016) and then explain the adaptations.

To extract the attended and ignored speech envelopes the audio data were first normalized, by dividing them by their standard deviation. Then, the absolute Hilbert transform was computed, and low-pass filtered at 8 Hz (Butterworth, filter order: 3). Lastly, the filtered data were down-sampled to 64 Hz to reduce subsequent computation times. In accordance with the two speech envelopes, the cEEGrid data were also low pass filtered at a cutoff frequency of 8 Hz (finite impulse response filter, Hann windows, filter order: 100), and then high-pass filtered at a cutoff frequency of 2 Hz (finite impulse response filter, Hann windows, filter order: 500). Afterward, the filtered cEEGrid data were normalized by dividing them by their standard deviation, and then down-sampled to 64 Hz.

For speech envelope tracking, we implemented a decoding model, i.e., we trained a model on an individual’s cEEGrid data to predict the attended speech envelope. For a better replicability, we implemented the decoding model within the mTRF toolbox (version 2.1; Crosse et al., 2016). For this, the individual’s cEEGrid data and speech envelopes were first segmented into non-overlapping 60 s segments using the mTRFpartition function. This resulted in 30 segments of each speech envelope and the corresponding cEEGrid data for each participant. Using the function mTRFattncrossval, a decoder was trained on 29 segments of the attended speech envelope and the corresponding cEEGrid data. This decoder was then used to reconstruct the attended speech envelope of the left-out segment. Afterward, the reconstructed speech envelope was correlated with the attended and ignored speech envelope of the left-out segment, respectively. The difference between these Pearson correlation coefficients (Corr_att_–Corr_ign_) is considered as the attentional gain. If the attentional gain was positive, the left-out segment was regarded as classified correctly. The prediction error was quantified as the mean squared error between the reconstructed and the actual speech envelope. The process of training a decoder on 29 segments and testing it on the left-out segment was repeated 30 times in a leave-one-out cross-validation manner (Stone, 1974). The decoding accuracy was then determined as the percentage of correctly classified segments. Chance level decoding accuracy was based on a binomial significance threshold.

In the decoding model, two important model hyperparameters require adjustment. One is the time lag window, which accounts for the time between the onset of the presented auditory stimulus and its cortical response. The other is the regularization parameter. Regularization is a technique to avoid overfitting and estimate reliable model parameters that generalize to unseen data (Holdgraf et al., 2017). Regularization is especially important in decoding models as it strongly affects the decoding accuracy (Wong et al., 2018). To closely follow the analysis pipeline used in Mirkovic et al. (2016), we applied Tikhonov regularization. Therefore, we estimated the optimal regularization parameter which we then multiplied with the regularization matrix (Crosse et al., 2016).

In line with Mirkovic et al. (2016), the optimal model hyperparameters, i.e., the time lag window and regularization parameter λ, were initially chosen on a group level. To this end, the grand average decoding accuracies were computed for different sets of hyperparameters. Potential time lag windows of 45 ms duration ranged from –115 to 620 ms, with 30 ms of overlap. Potential regularization parameters ranged from 10^–5^ to 10^5^ in factors of 10. As a result, for each participant we computed the decoding accuracy of 517 different sets of hyperparameters, based on 47 different time lag windows and 11 regularization parameters. We then selected the set of hyperparameters that yielded the largest grand average decoding accuracy. These group-level based hyperparameters were then used for all individual decoders. So far, we have described how we implemented the analysis pipeline as similarly used in Mirkovic et al. (2016). In the following two sections, we will explain the adaptations we made to explore the effect of artifact correction and individually chosen hyperparameters on the decoding accuracy.

##### Effect of Artifact Correction

The first adaptation was to include artifact correction into the analysis pipeline. As mentioned in Mirkovic et al. (2016), one possible reason for the low decoding accuracies was that no eye-, muscle- or movement-related artifacts were corrected for. Therefore, we investigated the effect of artifact correction on the decoding accuracy. To this end, we once performed artifact correction before the data were pass-band filtered between 2 and 8 Hz and compared it to the uncorrected data filtered between 2 and 8 Hz. To evaluate the impact of artifact correction, we compared the individual decoding accuracies between uncorrected and ASR-corrected data using a Wilcoxon signed rank test. To quantify how much data was modified by ASR and to what extent, we split the filtered data into consecutive 1-s segments and calculated the spectral power in the frequency range from 2 to 8 Hz. For each 1-s segment, we then averaged the spectral power over all channels and calculated the change in dB from uncorrected to ASR-corrected data.

##### Effect of Individually Chosen Hyperparameters

As a second adaptation we used individually chosen hyperparameters, instead of using group-level chosen hyperparameters. Specifically, individualizing the time lag window may help to increase decoding accuracies. As mentioned above, the time lag window accounts for the time between the stimulus onset and its cortical response. It is well known that cortical response lags vary across individuals (Lauter and Karzon, 1990), as can also be seen in Mirkovic et al. (2019). To the best of our knowledge, the effect of individualizing the regularization parameter for speech envelope tracking has not been investigated. Therefore, we also explored this adaptation. To select the optimal hyperparameters for each participant separately, we chose the set of hyperparameters which yielded the highest decoding accuracy for the individual. If multiple sets of hyperparameters fulfilled this criterion, we selected the one set among them which yielded the lowest prediction error. We then compared the individual decoding accuracies between the models using group-level and individually chosen hyperparameters with a Wilcoxon signed rank test.

Leave-one-out cross-validation (standard cross-validation) is a technique to train and test a model, such that the data which is used to train the model is different from the data which is used to test the model (Stone, 1974). Leave-one-out cross-validation is commonly applied in auditory attention decoding research (O’Sullivan et al., 2015). To compare our results to other studies, we also followed this approach when comparing the decoding accuracies of models using group-level or individually chosen hyperparameters. However, one aspect of this approach, which is sometimes neglected, is that when a model includes hyperparameters to be tuned, validating the model should be done on a yet another part of the data (Holdgraf et al., 2017). This procedure ensures that the selected hyperparameters do not only lead to high decoding accuracies on the data used to select them, but also on independent data. To account for this potential bias, we performed an additional analysis using nested cross-validation (Varma and Simon, 2006; Parvandeh et al., 2020). To this end, we first randomly selected 10 out of the 30 segments per participant for later validation of our model. The remaining 20 segments were then used in a leave-one-out cross-validation manner to find the optimal set of hyperparameters. Afterward, all these 20 segments were used to train the model with the selected set of hyperparameters. Finally, the model was validated by computing the decoding accuracy of the 10 initially left-out segments. This entire process was repeated 50 times so that at each iteration 10 different segments were randomly selected for later validation. In the end, the decoding accuracies were averaged over all 50 iterations. To test whether the results obtained in the initial analysis without independent validation data were biased, we performed the nested cross-validation approach once with group-level chosen and once with individually chosen hyperparameters. The difference between the resulting decoding accuracies was statistically evaluated using a Wilcoxon signed rank test.

#### Intersubject Correlations

Cap-EEG-based ISCs within a two competing speaker paradigm have previously been analyzed by Rosenkranz et al. (2021). In the current study, we performed the ISC analysis on the simultaneously acquired cEEGrid data. The aim was to test whether the attentional effect of ISCs, as observed by Rosenkranz et al. (2021), can also be observed with unobtrusive around-the-ear EEG recordings. Therefore, we closely followed the analysis pipeline presented in Rosenkranz et al. (2021), which was largely based on the publicly available code^4^ from Cohen and Parra (2016). As mentioned above, ISCs are based on the observation, that people who are exposed to the same stimulus show similar brain activity. In the current study, the first 10-min block was the only one which was included for all participants. Therefore, to leverage the statistical power of the entire sample size, for the ISC analysis we only used the first 10-min block. This was also done to closely follow the analysis performed in Rosenkranz et al. (2021).

To compute ISCs, the preprocessed cEEGrid data from the first 10-min block were first cleaned from artifacts as described above. After artifact correction the data were low-pass filtered at a cutoff frequency of 40 Hz (finite impulse response filter, Hann windows, filter order: 100), and then high-pass filtered at a cutoff frequency of 1 Hz (finite impulse response filter, Hann windows, filter order: 500). Lastly, the data were down-sampled to 250 Hz. Simply correlating the individual EEG channels between participants would not reveal a good estimate of the ISCs due to the low signal to noise ratio of EEG (Dmochowski et al., 2012). Therefore, Dmochowski et al. (2012) developed the correlated component analysis, which is available in the publicly available code from Cohen and Parra (2016). In the correlated component analysis, EEG channels are linearly projected such that the resulting components are maximally correlated between participants. Importantly, the number of resulting components is identical to the number of initial EEG channels. Lastly, the ISC scores of the three most correlating components were summed, resulting in a single ISC sum score per individual. Other components were neglected as their correlations have been shown to be close to chance (Ki et al., 2016).

##### Attentional Effect on Intersubject Correlations

When presented with two concurrent auditory streams, those participants attending to the same stream show higher ISC sum scores than those attending to different streams, even though all individuals are exposed to the same physical stimulus (Stuldreher et al., 2020; Rosenkranz et al., 2021). Here, we investigated whether this attentional effect could also be observed with cEEGrids. To test this, the cEEGrid data of each participant were correlated once with the cEEGrid data of all participants attending to the same audio book (ISC_same_) and once with the cEEGrid data of all participants attending to the other audio book (ISC_other_). Importantly, the projection vector of the correlated component analysis was computed on all but the to-be-correlated participant to reduce the risk of overfitting. For each participant, this resulted in 16 components (number of available cEEGrid channels) for the ISC_same_ condition and 16 components for the ISC_other_ condition. Within a participant, we then compared the ISC scores of the different components between the ISC_same_ and ISC_other_ condition. This we did for the three most correlating components individually as well as for their sum, using paired sample *t*-tests. The difference between an individual’s ISC sum score in the same and other condition is considered as the attentional effect (ISC_same_–ISC_other_). To compute the chance level for ISC scores, we created chance-distributions with circular time-shifted data (Parra et al., 2018). For each participant the data were shifted to a different extent but all EEG channels within a participant were shifted equally. This disturbed the temporal alignment between the participants’ EEG but kept the temporal and spatial structure within a participant unchanged. The process of randomly shifting the data and computing ISC scores for both conditions was repeated 100 times. This resulted in a distribution of ISC scores for each component and condition separately. The 95th percentiles of these distributions served as chance level.

In addition, we also classified whether a person attended to the left or right story based on their ISC scores (Rosenkranz et al., 2021). Therefore, we once computed the ISC scores of each participant with all participants attending to the left audio book (ISC_left_) and once with all participants attending to the right audiobook (ISC_right_). Thus, ISC_left_ and ISC_right_ reflect the synchrony of one participant with others attending to the left or right audio book, respectively. For this analysis we used two projection vectors, one was computed on participants who attended to the left story, and one was computed on participants who attended to the right story. Again, the to-be-correlated participant was left out when computing the projection vectors. Lastly, we summed the ISC scores of the three most correlating components and classified the direction of attention based on the ISC sum scores. Classification accuracy was calculated using the area under the receiver operator curve. Chance level accuracy was estimated by randomly assigning the class labels left and right and then calculating the corresponding area under the receiver operator curve (Ki et al., 2016). This was repeated 1000 times, each time randomizing the class labels. The 95th percentile of this distribution was then considered as chance level. Lastly, we evaluated the neurophysiological plausibility of the ISC components. For that, we computed the projection vectors of the correlated component analysis once for those participants attending to the left story, once for those attending to the right story and once for all participants. As the projection vectors are not directly physiologically interpretable, the projection vectors (spatial filters) were transformed into spatial patterns (Haufe et al., 2014).

#### Spectral Entropy

To compute the spectral entropy, the preprocessed cEEGrid data were first cleaned from artifacts as described above. Additionally, the function clean_channels was used to identify channels which correlated less than 0.6 with their robust estimate. These channels were later neglected when calculating the spectral entropy averaged over channels. Here, only for three participants one artifactual channel was identified. Spectral entropy was computed based on the analysis described in Lesenfants and Francart (2020). For each one-min segment and channel, the spectrum from 8 to 32 Hz was computed using multitaper spectral analysis (7 tapers, MATLAB function: pmtm). Each spectrum was then normalized by dividing each frequency power by the sum of all frequency powers in the range from 8 to 32 Hz (Viertiö-Oja et al., 2004). Thereby, the power of each individual spectrum was equalized to one which enabled the comparison between participants and channels. The spectral entropy was then computed as the product between the normalized frequency power of each frequency bin and the logarithm of its inverse. These were then summed over all frequency bins and normalized by one over the logarithm of the number of frequency bins. This resulted in spectral entropies ranging between zero and one. A spectral entropy value close to one reflects a spectrum in which the power of each frequency bin is similar, whereas a lower spectral entropy indicates a spectrum in which the power of the spectrum is concentrated in a few frequency bins. In the end, we had 30 (segments) times 16 (channels) spectral entropy values per participant. We did not have any prior assumptions on which cEEGrid channels to use. Therefore, we averaged the spectral entropy values over non-artifactual channels. Spectral entropy has been linked to the level of sustained attention, with higher values reflecting higher levels of attention (Lesenfants et al., 2018). There is both behavioral and neurophysiological evidence that the level of auditory attention decreases over time (Moore et al., 2017). Therefore, we investigated the spectral entropy over time by computing the Spearman rank correlation coefficient between the segment number and the corresponding spectral entropy. This we did for each participant individually as well as for the grand average spectral entropy. Alpha power (8–12 Hz) has also consistently been associated with attention (Foxe and Snyder, 2011; Klimesch, 2012). As it comprises one important frequency band when computing the spectral entropy based on the frequency spectrum from 8 to 32 Hz, we also investigated alpha power over time. For that we averaged the normalized frequency power from 8 to 12 Hz over all non-artifactual channels.

#### Relation Between Attentional Measures

To investigate the relation between the attentional gain in speech envelope tracking (Corr_att_–Corr_ign_) and the attentional effect of ISC sum scores (ISC_same_–ISC_other_), the time resolved attentional gain values in speech envelope were averaged over time. This resulted in one speech envelope gain value and one ISC sum difference score per participant. To investigate the relation between the attentional gain in speech envelope tracking (Corr_att_–Corr_ign_) and the spectral entropy we performed two analyses. Unlike Lesenfants and Francart (2020), we followed a correlation-based approach. In the first analysis, we correlated the time resolved speech envelope gain values with the time resolved spectral entropy values for each participant separately. In the second analysis, we first averaged the time resolved speech envelope gain and spectral entropy values over time to have one value pair per participant. We then correlated these value pairs for all participants. In all of the above-mentioned correlational analyses, the attentional gain of speech envelope tracking was computed using artifact corrected data and individual hyperparameters identified with standard cross-validation. Lastly, we correlated the attentional effect observed in the ISC sum scores (ISC_same_–ISC_other_) with the spectral entropy values averaged over time. To statistically evaluate the correlations, we performed Spearman rank correlations.

## Results

### Speech Envelope Tracking

**FIGURE 2:**
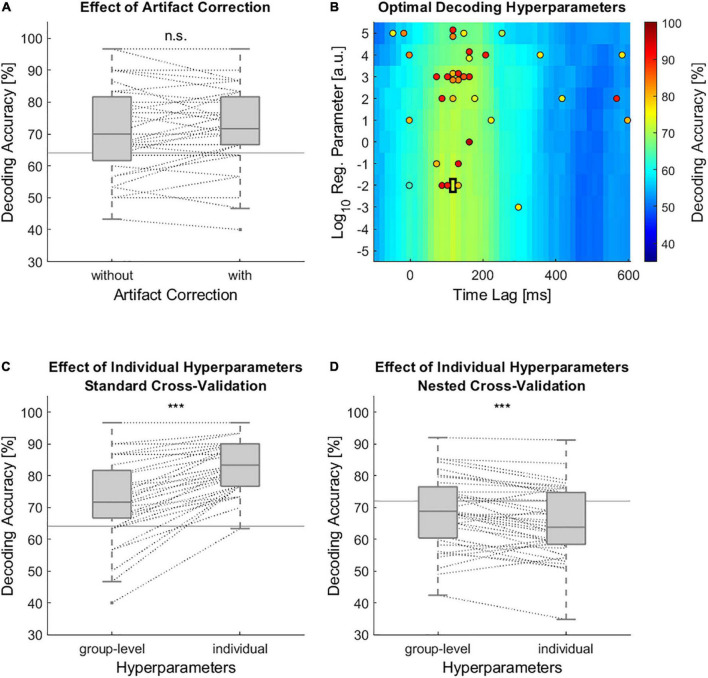
Effects on the accuracy of speech envelope decoding models. **(A)** Decoding accuracies of the individual models with and without artifact correction. In this analysis a group-level based time lag window from 95 to 140 ms and a regularization parameter of 10^−2^ were used for all individual models. **(B)** Decoding accuracies as a function of time lag window and regularization parameter. Black rectangle marks the group-level based optimal set of hyperparameters. Colored circles mark the optimal set of hyperparameters for each participant. The color within the circle indicates the decoding accuracy of a participant which resulted from using these hyperparameters. Due to an overlap of potential time lag windows only the center of a time lag window is displayed. **(C)** Decoding accuracies with group-level chosen and individually chosen hyperparameters. These decoding accuracies were based on standard leave-one-out cross-validation including 30 test trials. **(D)** Decoding accuracy with group-level and individually chosen hyperparameters based on nested cross-validation. Within the nested cross-validation only 10 test trials were used. **(A,C,D)** Horizontal gray lines indicate chance level decoding accuracy which were based on binomial significance thresholds. Dashed lines connect data points of the same participant (n.s. non-significant, *** *p* < 0.001).

When performing speech envelope tracking without artifact correction, the grand average decoding accuracies reached 71.3% (Figure 2A). Removing artifacts with ASR resulted in a grand average decoding accuracy of 72.13%, which was, however, not significantly higher (Figure 2A, Wilcoxon signed rank test, *Z* = 0.84, *p* = 0.4). In this analysis, the group-level chosen time lag window from 95 to 140 ms and a regularization parameter of 10^–2^ were used (Figure 2B, black rectangle). Most part of the data was not strongly modified by artifact correction. In fact, the change in spectral power (8–12 Hz) due to ASR was less than ±0.1 dB in 73.41% of all 1-s segments. In only 6.12% of all 1-s segments, the spectral power was changed more than ±3 dB (Supplementary Figure 1). Even though artifact correction did not significantly increase the decoding accuracy, all further analyses were performed on artifact corrected data to ensure that decoding the attended speaker is based on brain data and not on artifacts. Using individually chosen hyperparameters instead of group level chosen ones significantly increased the decoding accuracies to 82.59% (Figure 2C, Wilcoxon signed rank test, *Z* = 5.04, *p* < 0.001). The individually chosen optimal hyperparameters are shown in Figure 2B. However, when further controlling for overfitting with nested cross-validation the decoding accuracies dropped substantially and the group-level chosen hyperparameters outperformed those of individually chosen hyperparameters (Figure 2D, Wilcoxon signed rank test, *Z* = –3.88, *p* < 0.001).

### Intersubject Correlations

**FIGURE 3:**
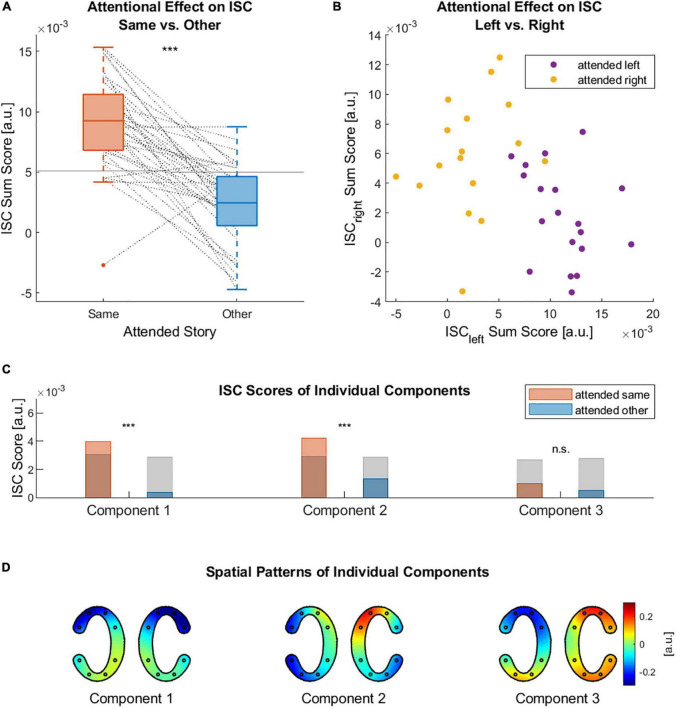
Attentional effects on ISC. **(A)** ISC sum scores of each participant with all those attending to the same story (ISC_same_) and with all those attending to the other story (ISC_other_). Horizontal lines indicate chance level based on circular time-shifted data. Dashed lines connect data points of the same participant. **(B)** ISC sums scores of each participant with all those attending to the left story (ISC_left_) and with all those attending to the right story (ISC_right_). **(C)** Grand average of the ISC scores of three strongest components. Once computed between those participants attending to the same story and once for those attending to different stories. Gray bar indicates chance level based on circular time-shifted data. **(D)** Spatial patterns (cEEGrid topographies) of the three strongest ISC components over all participants, independent of which story they attended to. In each pair of cEEGrids the left and right cEEGrid are depicted (n.s. non-significant, *** *p* < 0.001).

Using cEEGrid data, we confirmed the expected effect that ISC scores were significantly higher for participants attending to the same audio book than for those attending to different audio books (Figures 3A,C). This was the case for the ISC sum scores, i.e., the sum of ISC scores of the three strongest components (Figure 3A, paired sample *t*-test, *t* = 8.24, *p* < 0.001), as well as for the ISC scores of the first two components (Figure 3C, paired sample *t*-test, component 1: *t* = 7.93, *p* < 0.001, component 2: *t* = 6.2, *p* < 0.001). For the third component there was no evidence for a difference in ISC scores between the same and other conditions (Figure 3C, paired sample *t*-test, *t* = 1.5, *p* = 0.14). Only the ISC score of the first and second component revealed above chance level effects. The ISC sum scores of each individual participant with all those attending to the left and right audio book enabled us to classify to which audio book a participant was attending to Figure 3B. Classifying whether participants attended to the left story, using their ISC sum score with participants who attended to the left story, yielded an accuracy of 97.83%. Classifying whether participants attended to the right story, using their ISC sum score with participants who attended to the right story, yielded an accuracy of 80.05%. Both classification accuracies were clearly above chance level, which was at 65.94%. The spatial patterns of the condition-independent ISC components are shown in Figure 3D. We also provide the spatial patterns for all those participants attending to the left and those attending to the right in the Supplementary Material. Keeping in mind sign ambiguities, the spatial patterns of the left and right condition did not differ strongly from each other, nor from the condition-independent patterns (Supplementary Figure 2).

### Spectral Entropy

**FIGURE 4:**
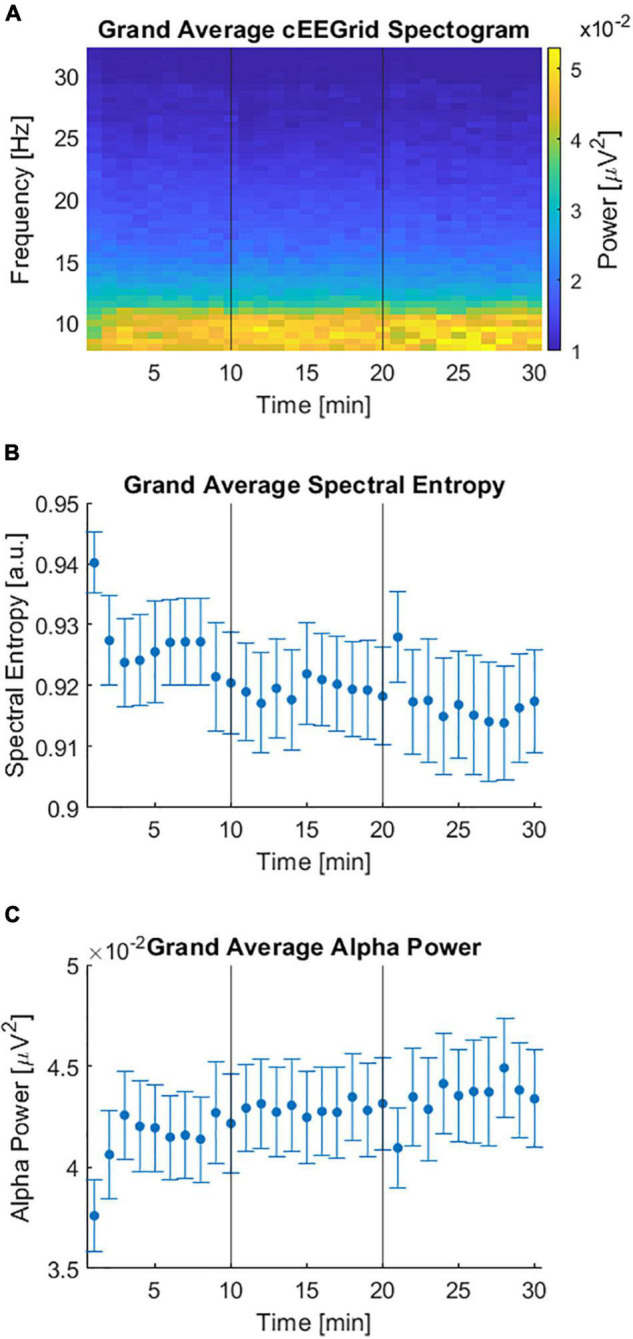
Spectral domain of cEEGrid data during the competing speaker paradigm. **(A)** Grand average spectrogram over all channels and participants in the frequency range from 8 to 32 Hz. **(B)** Spectral entropy over time averaged over channels and participants. Error bars reflect the standard error over participants. **(C)** Alpha power (8–12 Hz) over time averaged over channels and participants. Error bars depict the standard error over participants. **(A–C)** Vertical lines at 10 and 20 min indicate the end of a preceding 10-min block.

Figure 4A shows the average of all individual spectrograms from 8 to 32 Hz which in turn were averaged over all but the artifactual channels. The grand average spectral entropy decreased over time (Figure 4B, Spearman rank correlation, rho = –0.81, *p* < 0.001). On an individual level the spectral entropy significantly decreased over time for 12 participants while it significantly increased for 5 participants. For the remaining 19 participants there was no significant change over time (Supplementary Figure 3). In line with a decrease in the grand average spectral entropy, the grand average alpha power significantly increased over time (Figure 4C, Spearman rank correlation, rho = 0.82, *p* < 0.001).

### Relation Between Attentional Measures

**FIGURE 5:**
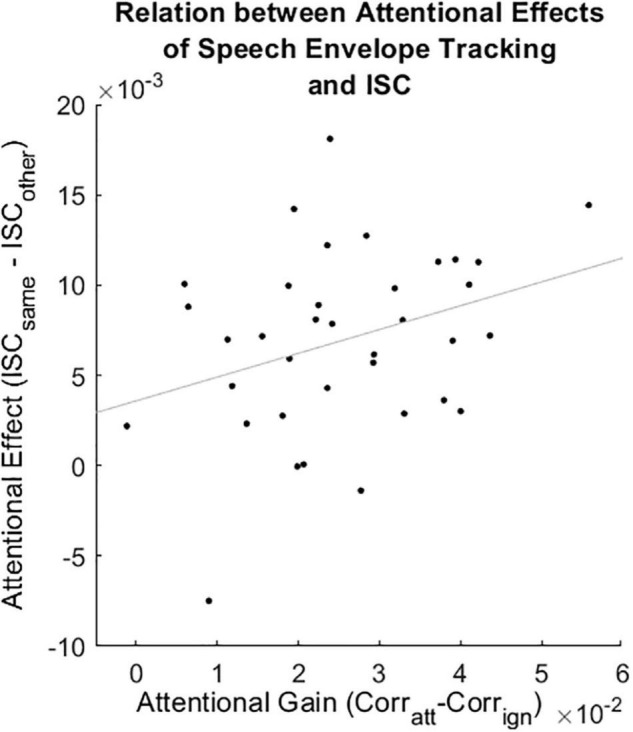
Correlation between the attentional gain in speech envelope tracking and the attentional effect in ISC sum scores (Spearman rank correlation, rho = 0.3, *p* = 0.04). Corr_att_: Spearman correlation between the predicted and the attended speech envelope. Corr_ign_: Spearman correlation between the predicted and the ignored speech envelope. ISC_same_: ISC sum score between a participant and all others attending to the same story. ISC_other_: ISC sum score between a participant and all others attending to the other story. Gray line represents the least square regression.

The attentional gain observed in speech envelope tracking (Corr_att_–Corr_ign_) correlated positively with the attentional effect observed in the ISC sum scores (ISC_same_–ISC_other_, Figure 5, Spearman rank correlation, rho = 0.3, *p* = 0.04). There was no evidence for a relation between the attentional gain observed in speech envelope tracking and the spectral entropy, neither for any individual participant nor for the time aggregated analysis (Supplementary Figure 4A, Spearman rank correlation, rho = –0.22, *p* = 0.19). There was no evidence for a relation between the attentional effect observed in ISC sum scores and the spectral entropy values averaged over time (Supplementary Figure 4B, Spearman rank correlation, rho = 0.06, *p* = 0.73).

## Discussion

Methods such as speech envelope tracking, ISCs, and spectral entropy help to analyze the neural processing of continuous stimuli. We show that all three methods capture complementary information about attention when the neural data is acquired exclusively with small flex-printed electrodes placed around the ear. Speech envelope tracking reliably decodes the attended of two concurrently presented speakers using cEEGrid data. We found that artifact correction did not increase the decoding accuracies while individualizing hyperparameters of the decoding models did. Moreover, ISCs based on cEEGrid data showed more similar brain activity between an individual with those attending to the same speaker than with those attending to another speaker. Regarding spectral entropy, we found that values obtained from cEEGrid data decreased over time, potentially reflecting a decrease in the participants’ level of attention. Interestingly, the attentional gain of speech envelope tracking and the attentional effect of ISC sum scores correlated positively while there was no evidence that either of these two measures correlated with the spectral entropy values.

### Speech Envelope Tracking

#### Effect of Artifact Correction

By approximating the analysis pipeline described in Mirkovic et al. (2016), the resulting decoding accuracies observed in the current study were comparable to Mirkovic et al. (2016). However, in contrast to our expectations, attenuating artifacts with ASR did not increase the decoding accuracy. One explanation could be that the short duration of typical artifacts only makes up a small portion of the 60 s segments that were used for decoding and thus artifact reduction does not strongly affect the decoding accuracy. In fact, only a small portion of the data contained strong artifacts which were corrected by ASR. Consequently, the decoding accuracy may benefit more from artifact correction when shorter segments of data are used. In Jaeger et al. (2020), ASR improved classification of shorter data segments, whereas Straetmans et al. (2022) showed that even for data segments as short as 5 s, decoding accuracies were not increased when the data was cleaned with ASR. We speculate that these heterogenous results could reflect the quality of the calibration data that were used to perform ASR. In Jaeger et al. (2020), the calibration data were extracted while participants performed a task (i.e., the competing speaker paradigm). In Straetmans et al. (2022), the calibration data were acquired while participants were seated without performing any task. It is known that good calibration data are crucial when performing ASR (Blum et al., 2019).

#### Effect of Individually Chosen Hyperparameters

We tested the effect of individualizing the classification model hyperparameters on the decoding accuracy using the commonly applied standard leave-one-out cross-validation. As could be expected, we observed higher decoding accuracies for models using individually chosen hyperparameters compared to models using group-level chosen hyperparameters. However, implementing standard leave-one-out cross-validation involves the risk of overfitting, since the same data are used for choosing the optimal hyperparameters and validating the model (Holdgraf et al., 2017). To account for this bias, we repeated the analysis using nested cross validation (Varma and Simon, 2006), where the validation of the model is done on a different part of the data than the training or the selection of hyperparameters (Parvandeh et al., 2020). In contrast to the results obtained with standard cross-validation, when implementing nested cross-validation, the models using group-level chosen hyperparameters outperformed those models using individually chosen hyperparameters. These results contradict studies showing that models fitted to the individual generally perform better than group-level based models (Mirkovic et al., 2015; O’Sullivan et al., 2015). Yet, when only a small amount of individual data is available, group-level based models outperform individualized models (Mirkovic et al., 2015). Only when a sufficient amount of data from an individual is supplied, does the individualized model outperform the group-level based model (Mirkovic et al., 2015). Therefore, we assume that a sufficiently large amount of individual data is necessary for the beneficial effect of individually chosen hyperparameters to become apparent when using nested cross-validation. Recently, a new approach has been proposed where the decoding models are initially provided with a participant-independent decoder which is then continuously updated as more data from the individual is available (Geirnaert et al., 2021a). To further investigate the effect of fitting the model to the individual, long-term recordings of an individual should be acquired. In contrast to cap-EEG acquisition, long-term data collection is certainly feasible with cEEGrids, providing good signal quality for many hours (Debener et al., 2015; Bleichner and Debener, 2017; Da Silva Souto et al., 2021; Hölle et al., 2021).

### Intersubject Correlation

We provide evidence that attentional effects of EEG-based ISCs can reliably be observed even when the neural data is recorded with a small number of electrodes placed around the ear. This is not a fully independent replication of the results reported by Rosenkranz et al. (2021), as the cap-EEG analyzed in that study was simultaneously acquired with the cEEGrid data presented here. However, it shows the potential of ear-EEG to measure attentional effects of ISCs. When comparing the ISC sum scores of cap-EEG with those of cEEGrid data, it becomes apparent that the cEEGrid based ISC sum scores are less often above chance. This is also the case for the ISC scores of the individual components. The fact that fewer ISC scores are above chance for cEEGrid data may be due to the lower number of channels and their spatial coverage. In fact, cEEGrid electrodes do not cover central parts of the scalp where ISCs are most prominently expressed (Rosenkranz et al., 2021). Nevertheless, the ISC sum scores were higher in the same than in the other condition for 33 out of 36 participants. In addition, the ISC_left_ and ISC_right_ sum scores enabled us to accurately classify to which audio book a participant attended to. These results demonstrate for the first time the sensitivity of around-the-ear EEG to attentional effects in ISCs.

It has been shown that attentional effects of ISCs can also be obtained based on other physiological data such as electrodermal or heartbeat activity, yet less reliably than based on EEG (Brouwer et al., 2019; Stuldreher et al., 2020; Pérez et al., 2021). However, in terms of application, electrodermal and heartbeat activity were preferred over traditional cap-EEG as those measures are easy to apply and cost efficient. Here we show that the cEEGrid presents a suitable candidate which fulfills both criteria–it can be used to obtain reliable attentional effects in ISCs, and it can be easily applied to unobtrusively measure one’s EEG. Thus, especially the combination of cEEGrids with cost-efficient data acquisition platforms such as the OpenBCI provide a setup that could be used for research in everyday life scenarios (Knierim et al., 2021). In addition, ISCs could also be based on a combination of EEG, electrodermal, and heartbeat activity, which has been shown to produce more accurate results than using EEG alone (Stuldreher et al., 2022).

### Spectral Entropy

The capacity to sustain attention in demanding tasks typically declines over time, coinciding with an increase in mental fatigue (Moore et al., 2017). Spectral entropy has been proposed as an objective measure of sustained attention (Lesenfants et al., 2018). In line with this, we found a decrease in spectral entropy over time. Since application of spectral entropy as a marker of sustained attention is a fairly new approach, there is limited evidence available to which we can compare our results. However, spectral entropy computed in the frequency range from 8 to 32 Hz strongly depends on alpha power (8–12 Hz), which in turn has also been associated with attention (Foxe and Snyder, 2011; Klimesch, 2012). The influence of alpha power on spectral entropy is evident in Lesenfants et al. (2018). They observed decreased alpha power and increased spectral entropy when participants were actively attending to a flickering stimulus compared to when the participants did not attend to the presented stimulus. We also found this inverse relation between alpha power and spectral entropy in the increase of alpha power over time. Such an increase in alpha power over time has been attributed to the depletion of attentional resources (Wascher et al., 2014). Furthermore, alpha band activity has been related to the suppression of task-irrelevant stimuli (Foxe and Snyder, 2011; Klimesch, 2012). Thus, the increase in alpha power and the decrease in spectral entropy might reflect the growing need to suppress the ignored speaker when mental fatigue accumulates.

### Relation Between Attentional Measures

We found a positive relation between the attentional gain in speech envelope tracking and the attentional effect of ISCs. This suggests that both measures reflect similar phenomena. Attended stimuli evoke a stronger neural response than ignored stimuli (Picton and Hillyard, 1974). While speech envelope tracking focuses on the aspect that the neural response toward the speech envelopes is consistent over time within a participant (Aiken and Picton, 2008), ISCs focus on the aspect that the neural response toward the same external stimuli is similar between participants, at least in sensory areas (Hasson et al., 2004).

Spectral entropy on the other hand is not directly linked to the neural response to the stimulus. When computing spectral entropy in the frequency range from 8 to 32 Hz, the lower frequencies, which are relevant for the brain to track the speech envelope (Giraud and Poeppel, 2012) are neglected. Spectral entropy may rather reflect a participant’s level of attention or vigilance (Lesenfants et al., 2018), that is, the capability to be aware and focus on external stimuli (van Schie et al., 2021). In contrast, speech envelope tracking and ISCs capture selective attention – the ability to select relevant and neglect irrelevant information. This may explain why we did not find a correlation between spectral entropy values and any of the two selective attention measures. This does not mean that one’s level of attention or vigilance does not influence one’s ability in selective attention, but only states there may not be a direct linear relation. In fact, Lesenfants and Francart (2020) showed that there is a difference in one’s selective attention ability during periods of high and low levels of attention/vigilance, but the exact nature of a potential relation between the selective attention and attention/vigilance needs to be further explored.

## Conclusion

The current study provides clear evidence that attentional measures to natural and continuous stimuli can be captured with around-the-ear EEG recordings, as provided with the cEEGrid. Ear-EEG opens up the possibility to capture neural traces of attentional processes unobtrusively in realistic everyday life scenarios. Future assistive devices could help those that have difficulties attending to one stream of information in the presence of distractor sounds.

## Data Availability Statement

The original cEEGrid contributions presented in the study are publicly available. These data can be found here: https://openneuro.org/datasets/ds004015. MATLAB code used to compute the results presented in the current study can be found on GitHub (https://doi.org/10.5281/zenodo.6379903).

## Ethics Statement

The studies involving human participants were reviewed and approved by Kommission für Forschungsfolgenabschätzung und Ethik, University of Oldenburg, Oldenburg, Germany. The patients/participants provided their written informed consent to participate in this study.

## Author Contributions

MJ and BH performed the data acquisition. BH analyzed the data and wrote the manuscript to which MR, MJ, SD, and BM contributed with critical revisions. All authors approved the final version and agreed to be accountable for this work.

## Conflict of Interest

The authors declare that the research was conducted in the absence of any commercial or financial relationships that could be construed as a potential conflict of interest.

## Publisher’s Note

All claims expressed in this article are solely those of the authors and do not necessarily represent those of their affiliated organizations, or those of the publisher, the editors and the reviewers. Any product that may be evaluated in this article, or claim that may be made by its manufacturer, is not guaranteed or endorsed by the publisher.

## Footnotes

1
https://github.com/labstreaminglayer

2
https://doi.org/10.5281/zenodo.6379903

3
https://doi.org/10.5281/zenodo.5946875

4
https://www.parralab.org/isc/
