# Supplementary material for: Ear-EEG Measures of Auditory Attention to Continuous Speech
Source: Front Neurosci. 2022 May 3;16:869426. doi: 10.3389/fnins.2022.869426 (PMC9111016; doi:10.3389/fnins.2022.869426)
Supplement: Supplementary file 1 [file Data_Sheet_1.docx]

Supplementary Material

# Supplementary Figures


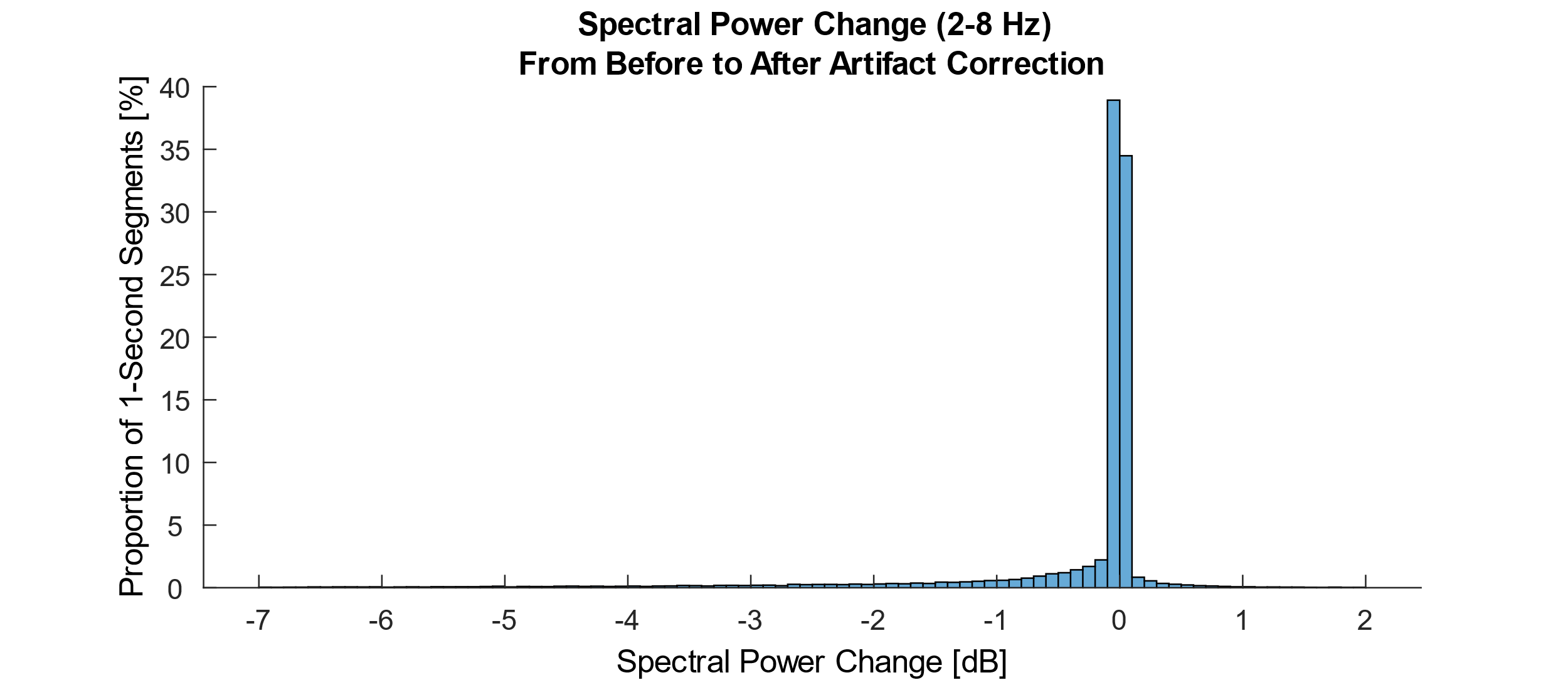


**Supplementary Figure 1** Proportion of data changed by artifact correction implemented with ASR. To compute the amount of data that was modified by ASR correction, we split the filtered data (2-8 Hz) into consecutive 1-second segments. For each segment, we then computed the spectral power in the frequency range from 2 to 8 Hz and averaged it over all channels. Here, we show how much of the data was influenced by ASR correction and by how much, computed as a change in spectral power from before to after artifact correction. For 73.41% of the segments the spectral power changed less than +/- 0.1 dB.


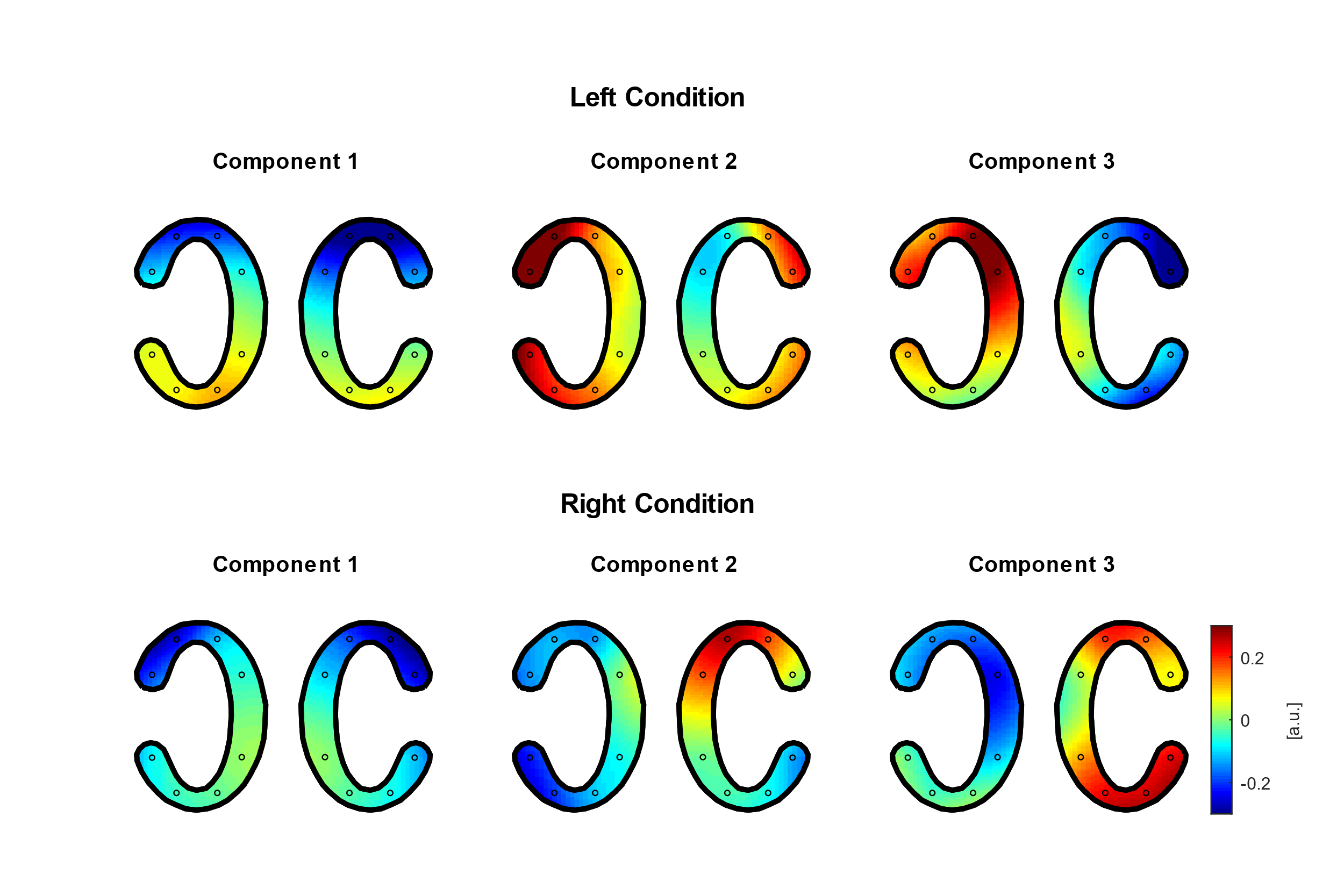

**Supplementary Figure 2** Spatial patterns of ISC components. (Top row) Spatial patterns of ISC components when only considering those participants attending to the left story. (Bottom row) Spatial patterns of ISC components when only considering those participants attending to the right story. In each pair of cEEGrids the left and right cEEGrid are depicted, respectively.


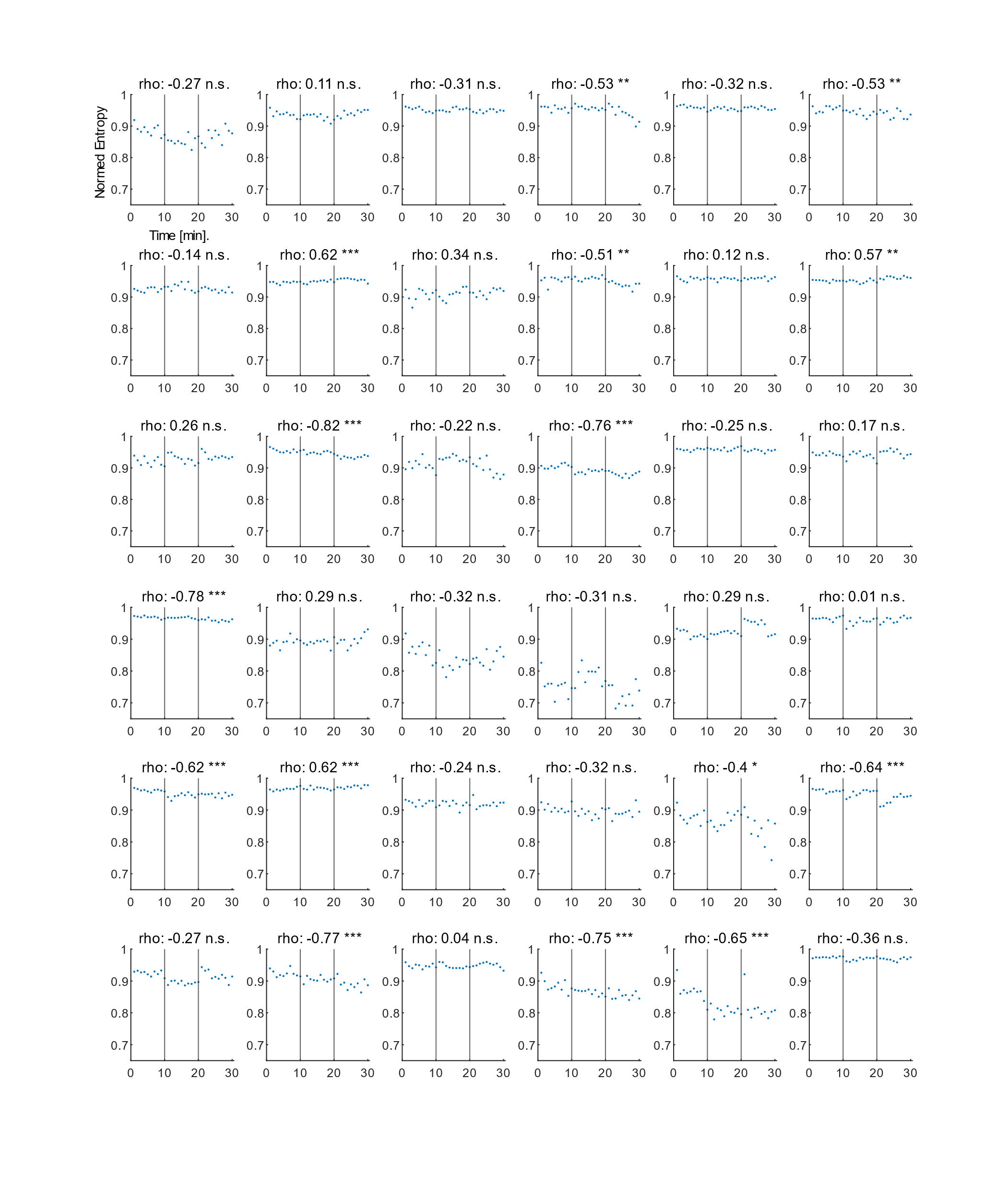

**Supplementary Figure 3** Spectral entropy over time for individual participants. Each subplot includes the data of a single participant. Spectral entropy values were averaged over all but the artifactual channels. The Spearman rank correlation between spectral entropy and time for each participant is depicted on top of each subplot. (n.s. non-significant, * p < 0.05, ** p < 0.01, *** p < 0.001).


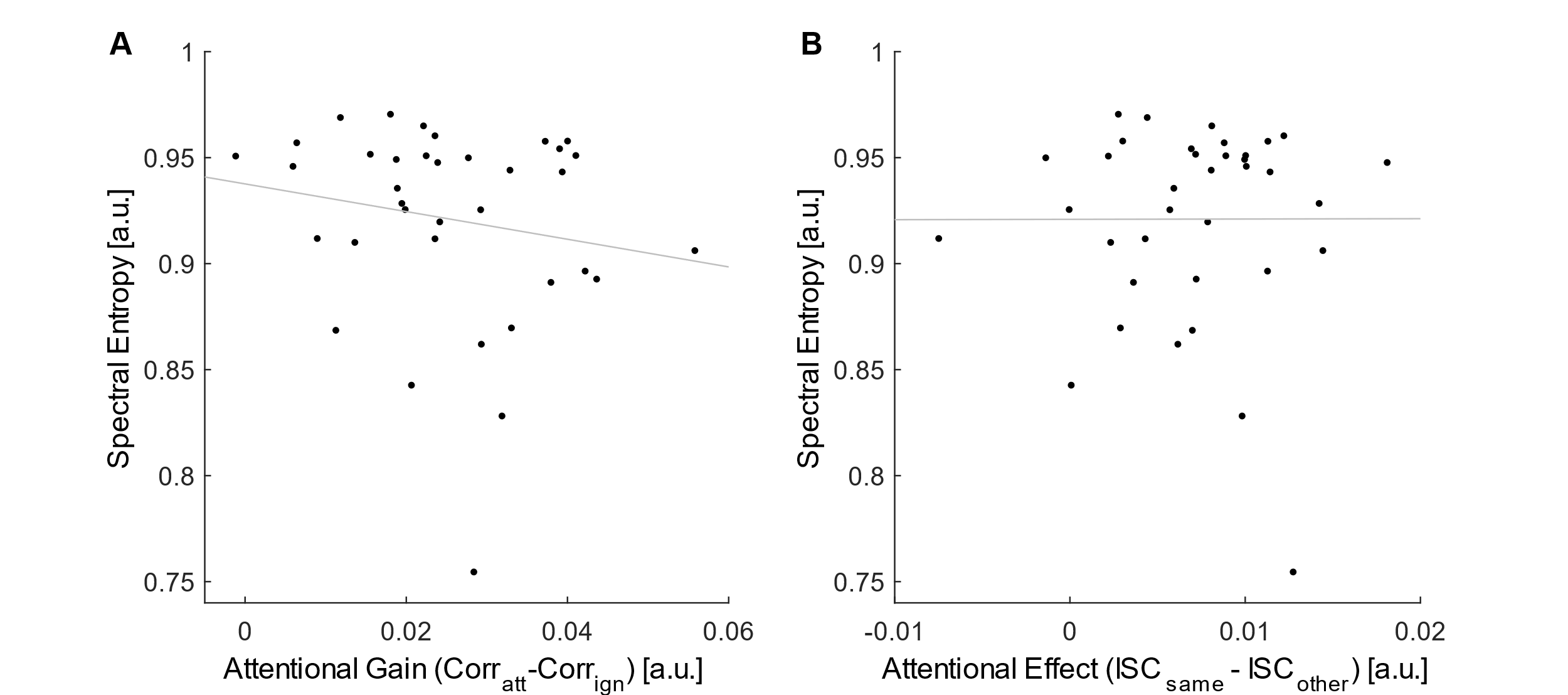


**Supplementary Figure 4** **(A)** Correlation between the attentional gain observed in speech envelope tracking and the spectral entropy (Spearman rank correlation, rho = -0.22, p = 0.19). For speech envelope tracking artifact correction was included. The decoding model was trained in a standard cross-validation procedure using individually chosen hyperparameters. Corr_att_: Spearman correlation between the predicted and the attended speech envelope. Corr_ign_: Spearman correlation between the predicted and the ignored speech envelope. Grey line represents the least square regression. **(B)** Correlation between the attentional effect observed in ISC sum scores and the spectral entropy (Spearman rank correlation, rho = 0.06, p = 0.73). ISC_same_: ISC sum score between a participant and all others attending to the same story. ISC_other_: ISC sum score between a participant and all others attending to the other story. Grey line represents the least square regression.
